# Supplementary material for: Correlates of diabetes mellitus and hypertension in India: Change as evidenced from NFHS- 4 and 5 during 2015–2021
Source: PLoS One. 2024 Jul 18;19(7):e0305223. doi: 10.1371/journal.pone.0305223 (PMC11257250; doi:10.1371/journal.pone.0305223)
Supplement: S1 File — They are numbered with captions as follows: Fig 1. Prevalence of DM (% wise) as per NFHS 4 and 5 in States across India. Fig 2. Prevalence of HTN across states (% wise) across India. Fig 3. Prevalence of both DM and HTN in same individuals (% wise) across the states in India.Fig 4. Self-Reported Disorders in female as per NFHS-4 and NFHS-5. Fig 5. Self-Reported Disorders in male as per NFHS-4 and NFHS-5. Table 1. NFHS-5 All means compared with ANOVA. Table 2. NFHS 4 All means compared with ANOVA. Table 3. Multivariate Regression analysis on Self-Reported Disorders. (DOCX) [file pone.0305223.s001.docx]

Supplementary Table 1: NFHS-5 All means compared with ANOVA .

|  | Sex | N | Mean | | | Std Dev | p value |
| --- | --- | --- | --- | --- | --- | --- | --- |
| All Data | Male | 101839 | 32.21 | | | 11.21 | 0.00 |
|  | Female | 724115 | 30.39 | | | 9.88 |  |
|  | Total | 825954 | 30.62 | | | 10.07 |  |
| Individuals Having HTN and T2DM | Male | 1553 | 44.12 | | | 7.99 | 0.00 |
|  | Female | 6682 | 41.13 | | | 7.10 |  |
|  | Total | 8235 | 41.69 | | | 7.37 |  |
| Individuals Having HTN | Male | 18826 | 38.53 | | | 10.08 | 0.00 |
|  | Female | 91419 | 37.01 | | | 8.99 |  |
|  | Total | 110245 | 37.27 | | | 9.20 |  |
| Individuals Having T2DM | Male | 3920 | 40.85 | | | 10.10 | 0.00 |
|  | Female | 19976 | | 37.64 | 9.13 | |  |
|  | Total | 23896 | | 38.17 | 9.37 | |  |

Supplementary Table 2. NFHS 4 All means compared with ANOVA .

|  | Sex | N | Mean | Std Dev | p value |
| --- | --- | --- | --- | --- | --- |
| All Data | Male | 112122 | 29.83 | 11.08 | 0.00 |
|  | Female | 699686 | 31.73 | 9.76 |  |
|  | Total | 811808 | 30.09 | 9.97 |  |
| Individuals Having HTN and T2DM | Male | 44.24 | 1299 | 7.43 | 0.00 |
|  | Female | 41.61 | 4637 | 6.36 |  |
|  | Total | 42.19 | 5936 | 6.69 |  |
| Individuals Having HTN | Male | 18903 | 37.74 | 10.07 | 0.00 |
|  | Female | 82887 | 36.20 | 9.05 |  |
|  | Total | 101790 | 36.48 | 9.27 |  |
| Individuals Having T2DM | Male | 3377 | 41.44 | 9.45 | 0.00 |
|  | Female | 13557 | 38.82 | 8.35 |  |
|  | Total | 16934 | 39.34 | 8.64 |  |

Supplementary Table 3: Multivariate Regression analysis on Self-Reported Disorders ( NFHS5)

|  | Diabetes  Values as OR (95%CI)  *denotes p value<.05 | | Hypertension  Values as OR (95%CI)  *denotes p value<.05 | | Both  (Values as OR (95%CI )  *denotes p value<.05 | |
| --- | --- | --- | --- | --- | --- | --- |
|  | NFHS 4 | NFHS 5 | NFHS 4 | NFHS 5 | NFHS 4 | NFHS 5 |
| Self reported heart disease | 6.35*  (5.98-6.74) | 6.21*  (5.78-6.67) | 2.29*  (2.19-2.39) | 1.00  (.91-1.10) | 4.23*  (3.81-4.69) | 1.28  (1.00-1.62) |
| Self Reported Asthma ‘ | 7.01*  (6.61-7.43) | 4.55*  (4.29-4.84) | 1.69*  (1.61-1.77) | 1.12*  (1.02-1.22) | 4.30*  (3.85-4.81) | 1.05  (0.68-1.61) |
| Self Reported Thyroid Disorder | 5.83*  (5.51-6.15) | 4.25*  (4.04-4.47) | 1.93*  (1.85-2.01) | 0.96  (0.87-1.06) | 5.02*  (4.56-5.52) | 0.84  (0.57-1.23) |
| Self Reported Cancer | 37.90*  (33.76-42.5) | 20.46*  (18.02-23.22) | 1.29*  (1.10-1.51) | 0.93  (1.00-0.92) | 8.77*  (6.90-11.13) | 1.07  (0.83-1.38) |

Supplementary figures 1-3 showing the state wise prevalence of DM , HTN and concurrent presence of both across states in India .

Figure 1


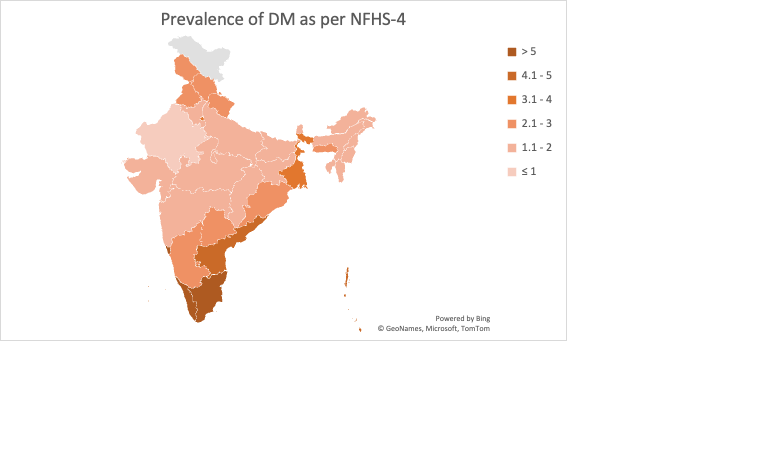


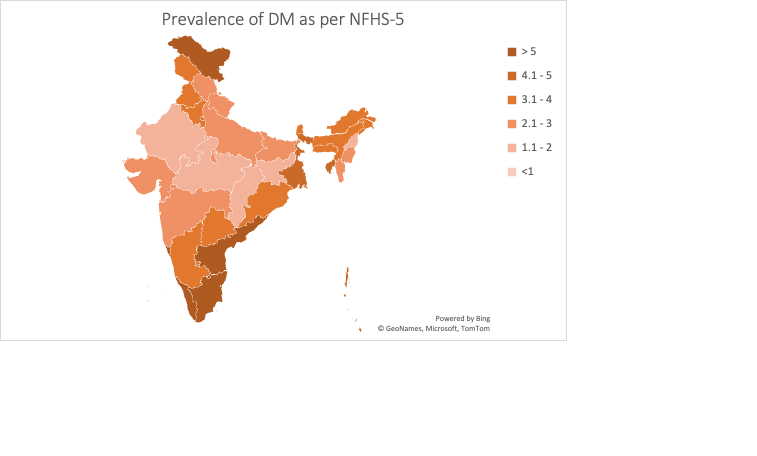


Figure 1. Prevalence of DM (% wise) as per NFHS 4 and 5 in States across India .

Figure 2


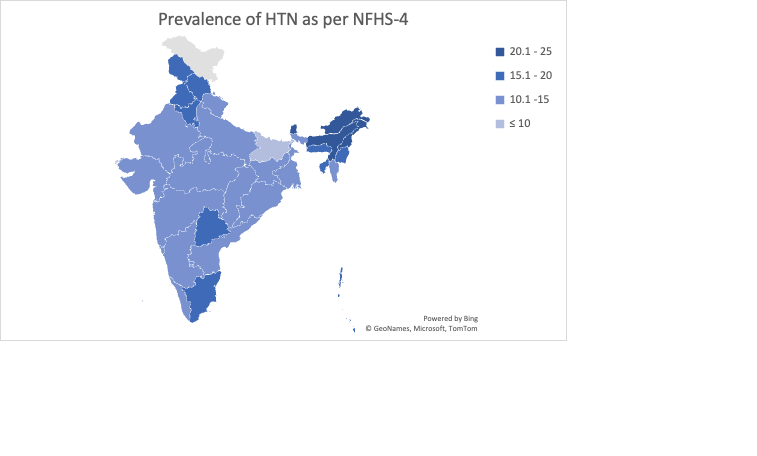


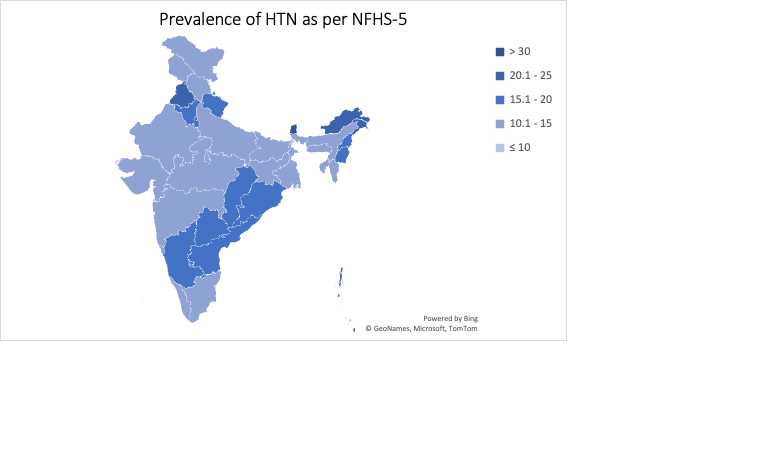


Figure 2 . Prevalence of HTN across states (% wise ) across India.

Figure 3. Prevalence of both DM and HTN in same individuals (% wise) across the states in India .

Figure 3


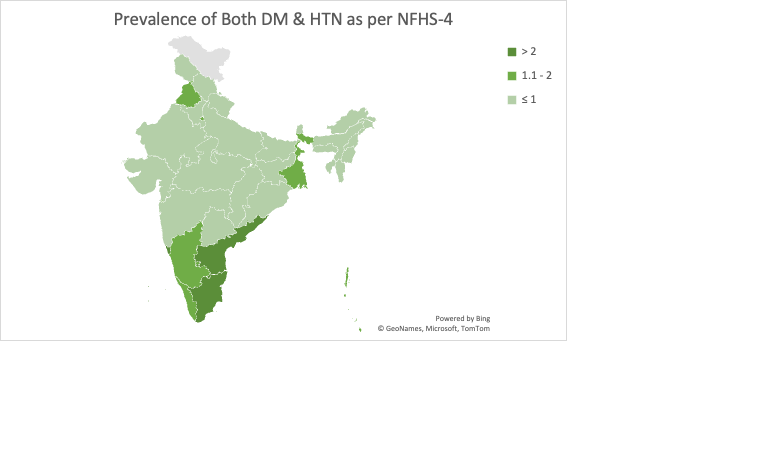


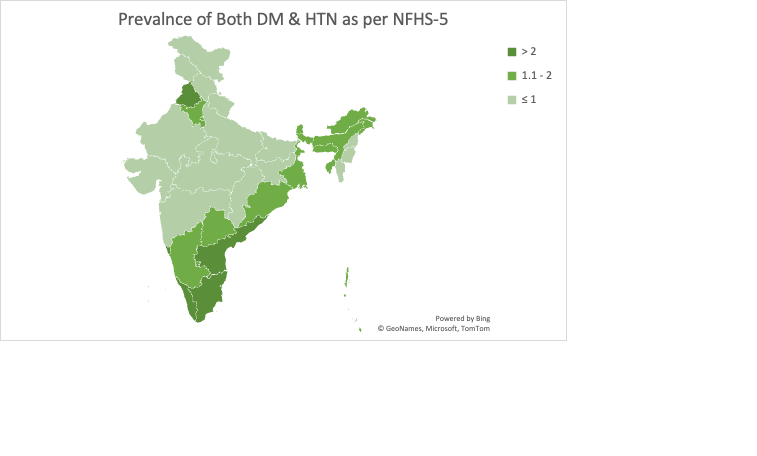


Figure 4: Self-Reported Disorders in female as per NFHS-4 and NFHS-5

Figure 5 :Self-Reported Disorders in male as per NFHS-4 and NFHS-5
