# Supplementary material for: Correlates of diabetes mellitus and hypertension in India: Change as evidenced from NFHS- 4 and 5 during 2015–2021
Source: PLoS One. 2024 Jul 18;19(7):e0305223. doi: 10.1371/journal.pone.0305223 (PMC11257250; doi:10.1371/journal.pone.0305223)
Supplement: Attachment — Submitted filename: PONE-D-23-28478_reviewer.pdf [file pone.0305223.s002.pdf]

PLOS ONE

Correlates of Diabetes Mellitus and Hypertension in India: Change as evidenced from NFHS- 4 and 5 during 2015-2021.

--Manuscript Draft--

**Manuscript Number:**	PONE-D-23-28478
**Article Type:**	Research Article
**Full Title:**	Correlates of Diabetes Mellitus and Hypertension in India: Change as evidenced from NFHS- 4 and 5 during 2015-2021.
**Short Title:**	Correlates of Diabetes Mellitus and Hypertension in India: NFHS 4 Vs 5.
**Corresponding Author:**	Sonu Goel, Ph.D.<br>University of Limerick<br>Limerick, IRELAND
**Keywords:**	NFHS-5, NFHS 4, Diabetes, Hypertension, Non-Communicable diseases, Cross referral, National data base
**Abstract:**	Objective: Both diabetes mellitus (DM) and hypertension (HTN) have been on the rise in recent decades all over the world more remarkably in developing countries like India. We intend to measure the prevalence of DM and HTN in the Indian population and to compare the trends and various correlates of these diseases in NFHS (National Family Health Survey)-4 and 5.Methodology: Country data of NFHS-4 and 5 were used.Total numbers of observations in individual and men recode files were 724115 and 101839 respectively. While for NFHS 4 the total numbers were for females as 699686 and for males 112122. All statistical analyses were done in SAS (version 9.4). Mixed effects survey logistic regression models were used for estimating odds ratio (OR) .p-values <0.05 were considered significant .Results: 1,637,762 individual case entries were evaluated. Both the diseases showed an increasing trend as per the advancing age in both sexes. The highest prevalence of DM was seen in the age group of 45 – 49 years (7.8%) in females and > 50 years (11.9%) in males as per NFHS-5. Similarly, the highest prevalence of HTN was seen in the age group of 45 – 49 years (31.2%) in females and > 50 years (41.4%) in males as per NFHS-5. The OR (95% CI) of prevalence of DM, HTN and both the diseases in age group >50 years was 14.46 (13.14-15.7), 16.65 (15.78-17.6), 79.5 (64.76-97.73) respectively when compared to reference age group15-19 years. Highest odds for having both DM, HTN concurrently was in age >50 years with aOR(95% CI) 65.32 (52.26-72.63) in NFHS 4 and 35.57 (97.47-45.53) in NFHS 5.Rise in prevalence of DM, HTN and concurrent presence is noted with an apparent increase in cases.
**Order of Authors:**	Rishabh Kumar Rana, MBBS , MD
	Ravi Ranjan Jha, MBBS , MD
	Ratnesh Sinha, MBBS , MD
	Dewesh Kumar, MBBS, MD , DNB
	Richa Jaiswal, MBBS , MSc
	Urvish Patel, MBBS , MPH
	Jung Bahadur Prasad, Msc , MPH
	Sitanshu Kar, MBBS, MD , MPH
	Sonu Goel, Ph.D.
**Additional Information:**	
**Question**	**Response**
**Financial Disclosure**<br><br>Enter a financial disclosure statement that	No funding received

describes the sources of funding for the work included in this submission. Review the submission guidelines for detailed requirements. View published research articles from *PLOS ONE* for specific examples.<br><br>This statement is required for submission and **will appear in the published article** if the submission is accepted. Please make sure it is accurate.<br><br>**Unfunded studies**<br>Enter: *The author(s) received no specific funding for this work.*<br><br>**Funded studies**<br>Enter a statement with the following details:<br>• Initials of the authors who received each award<br>• Grant numbers awarded to each author<br>• The full name of each funder<br>• URL of each funder website<br>• Did the sponsors or funders play any role in the study design, data collection and analysis, decision to publish, or preparation of the manuscript?<br>• **NO** - Include this sentence at the end of your statement: *The funders had no role in study design, data collection and analysis, decision to publish, or preparation of the manuscript.*<br>• **YES** - Specify the role(s) played.<br><br>* typeset	
**Competing Interests**<br><br>Use the instructions below to enter a competing interest statement for this submission. On behalf of all authors, disclose any competing interests that could be perceived to bias this work—acknowledging all financial support and any other relevant financial or non-financial competing interests.<br><br>This statement is required for submission and **will appear in the published article** if the submission is accepted. Please make sure it is accurate and that any funding	No conflicting interest

sources listed in your Funding Information later in the submission form are also declared in your Financial Disclosure statement.<br><br>View published research articles from *PLOS ONE* for specific examples.<br><br>**NO authors have competing interests**<br><br>Enter: *The authors have declared that no competing interests exist.*<br><br>**Authors with competing interests**<br><br>Enter competing interest details beginning with this statement:<br><br>*I have read the journal's policy and the authors of this manuscript have the following competing interests: [insert competing interests here]*<br><br>* typeset	
**Ethics Statement**<br><br>Enter an ethics statement for this submission. This statement is required if the study involved:<br><br>• Human participants<br>• Human specimens or tissue<br>• Vertebrate animals or cephalopods<br>• Vertebrate embryos or tissues<br>• Field research<br><br>Write "N/A" if the submission does not require an ethics statement.<br><br>General guidance is provided below. Consult the submission guidelines for detailed instructions. **Make sure that all information entered here is included in the Methods section of the manuscript.**	In this study, secondary analysis was done of NFHS-4 and 5 data sets. These data setswere available in the public domain from the DHS Program website and were de-identified.13The study was ethically approved by the Institute’s Ethical Committee, Postgraduate Institute of Medical Education and Research (PGIMER), Chandigarh (IEC-08/2022-2535 dated 17.08.2022).

**Format for specific study types**<br><br>**Human Subject Research (involving human participants and/or tissue)**<br>• Give the name of the institutional review board or ethics committee that approved the study<br>• Include the approval number and/or a statement indicating approval of this research<br>• Indicate the form of consent obtained (written/oral) or the reason that consent was not obtained (e.g. the data were analyzed anonymously)<br><br>**Animal Research (involving vertebrate animals, embryos or tissues)**<br>• Provide the name of the Institutional Animal Care and Use Committee (IACUC) or other relevant ethics board that reviewed the study protocol, and indicate whether they approved this research or granted a formal waiver of ethical approval<br>• Include an approval number if one was obtained<br>• If the study involved *non-human primates*, add *additional details* about animal welfare and steps taken to ameliorate suffering<br>• If anesthesia, euthanasia, or any kind of animal sacrifice is part of the study, include briefly which substances and/or methods were applied<br><br>**Field Research**<br><br>Include the following details if this study involves the collection of plant, animal, or other materials from a natural setting:<br>• Field permit number<br>• Name of the institution or relevant body that granted permission	
**Data Availability**<br><br>Authors are required to make all data underlying the findings described fully available, without restriction, and from the time of publication. PLOS allows rare exceptions to address legal and ethical concerns. See the PLOS Data Policy and FAQ for detailed information.	Yes - all data are fully available without restriction

A Data Availability Statement describing where the data can be found is required at submission. Your answers to this question constitute the Data Availability Statement and **will be published in the article**, if accepted.<br><br>**Important:** Stating 'data available on request from the author' is not sufficient. If your data are only available upon request, select 'No' for the first question and explain your exceptional situation in the text box.<br><br>Do the authors confirm that all data underlying the findings described in their manuscript are fully available without restriction?	
**Describe where the data may be found in full sentences. If you are copying our sample text, replace any instances of XXX with the appropriate details.**<br><br>• If the data are **held or will be held in a public repository**, include URLs, accession numbers or DOIs. If this information will only be available after acceptance, indicate this by ticking the box below. For example: *All XXX files are available from the XXX database (accession number(s) XXX, XXX.)*.<br>• If the data are all contained **within the manuscript and/or Supporting Information files**, enter the following: *All relevant data are within the manuscript and its Supporting Information files.*<br>• If neither of these applies but you are able to provide **details of access elsewhere**, with or without limitations, please do so. For example:<br><br>*Data cannot be shared publicly because of [XXX]. Data are available from the XXX Institutional Data Access / Ethics Committee (contact via XXX) for researchers who meet the criteria for access to confidential data.*<br><br>*The data underlying the results presented in the study are available from (include the name of the third party*	All .SAV files are available from the https://dhsprogram.com/data/Using-DataSets-for-Analysis.cfm#CP_JUMP_14039database (accession number(s)

and contact information or URL).<br>• This text is appropriate if the data are owned by a third party and authors do not have permission to share the data.<br><br>* typeset	
Additional data availability information:	Tick here if the URLs/accession numbers/DOIs will be available only after acceptance of the manuscript for publication so that we can ensure their inclusion before publication.; Tick here if your circumstances are not covered by the questions above and you need the journal's help to make your data available.

Manuscript with Title page
Click here to access/download;Manuscript;Manucript_nfhs_30.08.23.docx

Title of the study: Correlates of Diabetes Mellitus and Hypertension in India: Change as evidenced from NFHS- 4 and 5 during 2015-2021.

Authors and Affiliations in sequence

1. ***Dr Rishabh Kumar Rana***
MBBS, MD, Assistant Professor, Department of PSM, SNMMCH, Dhanbad, Jharkhand, India
Email bakwasandsony@gmail.com

2. ***Dr Ravi Ranjan Jha***
MBBS , MD , Assistant Professor , Department of PSM , SNMMCH , Dhanbad , Jharkhand , India
Email drravirjha@gmail.com

3. ***Dr Ratnesh Sinha***
MBBS , MD Community Medicine, Assistant Professor, Department of Community Medicine, Manipal Tata Medical College, Jamshedpur, Manipal Academy of Higher Education, Manipal, Karnataka, India - 576104
Email ratnesh2806@gmail.com

4. ***Dr Dewesh Kumar,***
MBBS, MD, Assistant Professor , Department of PSM , RIMS , Ranchi, Jharkhand , India
Email dr.dewesh@gmail.com

5. ***Dr Richa Jaiswal,*** MD, MSCR Clinical Research Assistant, Department of Emergency Medicine , Medical University of South Carolina, South Carolina, USA.
Email jaiswal@musc.edu

6. ***Dr Urvish Patel***, MD, MPH, Department of Public Health and Neurology, Icahn School of Medicine at Mount Sinai, NY, USA.
Email drurvishpatel@gmail.com

7. ***Dr Jung Bahadur Prasad*** , MPH , PhD, Professor Associate , KLE University Belagavi, Karnataka 590010. Email jbiips12@gmail.com

8. **Prof Dr Sitanshu Sekhar Kar** , MD, PhD, Professor and Head , Department of Community Medicine , JIPMER , Pondicherry , India Email - drsitanshukar@gmail.com

9. ***Prof Dr Sonu Goel , MBBS , MD , PhD Community Medicine and School of Public Health , PGI Chandigarh : Adjunct Professor University of Limerick Ireland .**

***Corresponding author**

***Prof Dr Sonu Goel** , MBBS , MD , PhD Community Medicine and School of Public Health ,

Email – sonu.goel@ul.ie

What is already known on this topic – India shares a large burden of cardiac and other related disease cause mortality to the rise in number of individuals suffering from Diabetes and Hypertension .

What this study adds – We were able to highlight different states and sections of the society where changes in the prevalence of both diseases are rising in a country data set .

How this study might affect research, practice or policy - summarise the implications of this study- This study will help in policy decision making by rationalising the priorities based on the trends of change . Resources could be directed where prevalence is more and more focus on preventive aspects can be summarily designed with focus on geolocation strategically .

Abstract

Both diabetes mellitus (DM) and hypertension (HTN) have been on the rise in recent decades all over the world more remarkably in developing countries like India. We intend to measure the prevalence of DM and HTN in the Indian population and to compare the trends and various correlates of these diseases in NFHS (National Family Health Survey)-4 and 5.Country data of NFHS-4 and 5 were. All statistical analyses were done in SAS (version 9.4). Mixed effects survey logistic regression models were used for estimating odds ratio (OR) .p-values <0.05 were considered significant .1,637,762 individual case entries were evaluated. Both the diseases showed an increasing trend as per the advancing age in both sexes. The highest prevalence of DM is seen in the age group of 45 – 49 years (7.8%) in females and > 50 years (11.9%) in males as per NFHS-5. Similarly, the highest prevalence of HTN was seen in the age group of45 – 49 years (31.2%) in females and > 50 years (41.4%) in males as per NFHS-5. The OR (95% CI) of prevalence of DM, HTN and both the diseases in age group >50 years was 14.46 (13.14-15.7), 16.65 (15.78-17.6), 79.5 (64.76-97.73) respectively when compared to reference age group15-19 years. Highest odds for having both DM, HTN concurrently was in age >50 years with aOR(95% CI) 65.32 (52.26-72.63) in NFHS 4 and 35.57 (97.47-45.53) in NFHS 5.Rise in prevalence of DM, HTN and concurrent presence is noted with an apparent increase in cases.
Keywords: NFHS-5, NFHS 4, Diabetes, Hypertension, Non-Communicable diseases, Cross referral

Background

In recent decades, diabetes mellitus (DM) has risen to prominence as one of the most pervasive and devastating diseases of our day, with the potential to shorten one's life span, render them disabled, and drain their finances. The 9th edition of the International Diabetes Federation (IDF) reported in 2019 that the worldwide prevalence of DM has reached pandemic proportions, with 9% (463 million individuals) affected. Recent estimates for prevalence estimates for those aged 20-79 show that worldwide rates of DM will reach 10.5% (536.6 million) in 2021 and 12.2% (783.2 million) in 2045.[1,2]

Hypertension (HTN) is a leading cause of disability-adjusted life years (DALYs) in men and women worldwide. Hemorrhagic and ischemic strokes and high systolic blood pressure are the biggest risk factors for ischemic heart disease.[3]HTN and DM are increasingly common and their interaction has been dubbed "bad companions." They mutually influence. Even with anti-HTN medication, uncontrolled blood pressure increases DM risk. Type 2 diabetics have a three-fold higher risk of HTN than non-diabetics. 50%–80% of type 2 DM patients have elevated blood pressure.[4–6]India is arguably the diabetic capital of the world, and cardiovascular diseases (CVDs) affected 14.1% of disability-adjusted life years (DALY) in 2016, up from 6.9% in 1990.[7,8] To establish an effective public health response and services system to manage the rise of these diseases, we need a clear understanding of the above scenario based on the local factors responsible for it. NFHS (National Family Health Survey) and DLHS (District level Household Survey) databases, which are indigenous and nationwide, have been studied in limited instances. [8,9]According to NFHS 4 data, 11.3 percent of Indians have HTN, 13.6 percent of males and 8.8 percent of females aged 15–54. [7]

The urban-rural HTN prevalence gap is smaller than previously reported. As the lifestyle has changed over time so has the spread of HTN over time.

2015–2016 NFHS 4 had 642344 females and 112122 boys. District Household Survey, a 2014 large sample database, is comparable to NFHS 4. Both surveys reported HTN prevalence at 25.3% (95% CI 25.0–25.6%), with men having a higher frequency (27.4%, CI 27.0–27.7%) than women (23.3–23.8%). [10]The rule of halves is internationally known, yet most large data set studies, like the NFHS 4 study or the recently published 10,000-person National Noncommunicable Disease Monitoring Survey (NNDMS), have not shown its application in India.[11]Amarchand R et al. (2022) used indigenous data from NFHS 4, DLHS 4, and AHS to find that 1.5% (0.76-2.96) of non-pregnant people had co- presence of high blood sugar and blood pressure. [12]

India is progressing rapidly, thus we need more recent data for planning. Analyzing the data of NFHS and comparing the traits of HTN will enable us to better understand the changing pattern of the disease across the states, age groups, and other societal statuses. This information can be used to draw a state-wise prioritization map for a better-rationalized approach in the country. From recent larger country data, we intend to have a better picture of the coexistence of HTN and DM. Our objectives of the study are to know the sociodemographic traits of the survey population , know the prevalence of DM,HTN and their co-existence in this population. We also will look for correlates of the various factors which could be responsible for these conditions in the population surveyed .

Methodology

In this study, secondary analysis was done of NFHS-4 and 5 data sets. These data setswere available in the public domain from the DHS Program website and were de-identified.[13]The study was ethically approved by the Institute's Ethical Committee, Postgraduate Institute of Medical Education and Research (PGIMER), Chandigarh (IEC-08/2022-2535 dated 17.08.2022). We used the most recent round of fifth National Family Health Survey (NFHS-5), conducted in 2019–20, which collected data on India's population's health and nutrition. IIPS Mumbai coordinated this multi-round, nationwide survey for the Ministry of Health and Family Welfare.Information regarding several risk factors of HTN was added to the NFHS-5 survey, which was absent from the NFHS-4 survey.[10]

Stratified random sampling was performed at multiple stages. A two-stage sampling design was used to select the rural sample. In the first stage, villages served as the primary sampling units, and they were chosen using a probability that was proportional to their population size. In the second stage, a random selection was made from among the 22 households that made up each primary sampling unit. In metropolitan regions, there was also a two-stage sample design that included selecting census enumeration blocks (CEB) in the first step and then selecting 22 households at random from each CEB in the second stage. Interviews were conducted with any woman or man in the sample families between the ages of 15 and 49 or 15 and 54. The national report of the NHFS-5 contains exhaustive information regarding the sampling procedures that were utilized for the survey. [10]

Data was collected from NFHS-5 individual recodes file (IAIR7AFL, which represented data for females) and the men's recodes file (IAMR7AFL). Total numbers of observations in individual and men recode files were 724115 and 101839 respectively. While for NFHS 4 the total numbers were for females as 699686 and for males 112122. In this study, we defined 'self-report diabetes' as all non-pregnant individuals who answered "yes" to the question "Do you currently have diabetes?". We defined 'undiagnosed diabetes' as those participants who answered 'no' to the question "do you currently have diabetes" and following a laboratory assessment either had an opportunistic fasting glucose level ≥126 mg/dL (referred to as 'fasting') or had a random glucose level ≥200 mg/dL (referred to as 'random'). Hypertension was defined as having a BP of 140/90 or responding "yes" to at least 1 of the 2 following questions: (a) "Were you told on 2 or more different occasions by a doctor or other health professional that you had hypertension or high blood pressure?" and (b) "To lower your blood pressure, are you now taking a prescribed medicine?"Concurrent presence of both in individuals who have both HTN and DM.

Independent variables studied were sex, age, religion, age groups (in 5-year bins), number of household members (total listed), occupation, wealth index (poorest, poor, middle, rich, and richest), level of education (none, primary, secondary, and higher), body mass index (bmi) (kg/m2), waist /hip ratio, smoking, drinking alcohol, place of residence (urban vs rural) and state of residence.While Comorbid conditions were self-reported heart disease, self-reported asthma and self-reported thyroid disorder.
All statistical analyses were performed by using the weighted survey methods in SAS (version 9.4) and p-value of <0.05 was considered to be significant. After assessing the parametricity of the data univariate analysis of differences between categorical variables was tested by using the chi-square test and analysis of differences between continuous variables (systolic and diastolic blood pressure, waist circumference, hip circumference, etc) was tested by using unpaired Student's t-test. Mixed-effects survey logistic regression models with

weighted analysis were used for the categorical dependent variables, including HTN, DM, and concurrent presence of both HTN & DM for the outcome of interest, in order to estimate the odds ratio (OR) and 95% Confidence Interval for the association between various independent variables in the NFHS 4 and 5. Multivariate logistic regression was performed to estimate adjusted odds ratios with their 95% CI with every measurement, prevalence of HTN, DM, and concurrent presence of both HTN & DM as dependent variables and the above variables as independent.

Results

It was noted that the burden of disease has increased in almost all the variables in NFHS-5 as compared to NFHS-4.A higher prevalence of DM, HTN, and Both diseases (HTN and DM) is seen with the increase in age in both genders. This trend is shown in both NFHS-4 and NFHS-5 data also we can see that the prevalence of the disease is more in NFHS-5 data as compared to NFHS-4 data.The highest prevalence of DM is seen in the age group of 45 – 49 years in females and > 50 years in males, richest wealth index, BMI > 30, urban residents, widowed participants, and having a history of alcohol consumption (Table 1) . A similar trend is shown for HTN and Both diseases (HTN and DM) (Tables 2 and 3). The overall prevalence of DM in females and males is 2% and 3.2% respectively as per NFHS-4 while in NHFS-5 it is 2.9% and 4.2% females and males respectively and an increase of 0.9% in females while 1% for males across the country (Table 1). Its prevalence rose from 13.2% to 13.5% in females and 18.7% to 21.6% in f males respectively as per NFHS 4 and NFHS 5 data. (Table 2)

Co presence of DM and HTN in females grew from 0.8% to 1.0% while in males from 1% to 1.8%. The age group of 50-54 years in males was the major contributor to this morbid condition co-presence of both the non-communicable lifestyle diseases. (Table 3), It was seen that mean ages for DM, HTN and co-presence of both was lower in NFHS-5 when compared with NFHS-4 with high statistical significance. (Table 1, 2 in Supplementary Files)

Multivariate logistic regression analysis was performed for estimating the odds of having DM, HTN, and both depending on various factors as per Tables 1, 2, and 3. Unadjusted Odds Ratio are presented in Table 4. Odds of having DM, HTN or their co-presence were seen to be less if one resided in rural areas and as one got educated. Higher odds of having these diseases were found to be in these predictors a) Age group as age grew when compared with 15-19 age group the risk of having these conditions grew with max in the high age group of 50-54. These odds were following the same pattern in both NFHS-4 and NFHS-5 and very high statistical significance. As body mass index grows and crosses 25 odds start to increase

for the occurrence of these diseases and their co-presence. Waist Hip Ratio an addition for NFHS-5 follows the accepted norm of having higher odds of having DM, HTN or both. When compared to Waist Hip Ratio <=1 the odds are 2.76 times more of having the co-presence of DM and HTN. Seen in both NFHS-4 and NFHS-5 is a protective trend in the case of education as compared to uneducated. As one tends to get a higher degree the odds are on the lower side for the occurrence of these conditions. Marriage is an important predictor for the presence of these conditions when compared to never married. It was seen that the highest odds of having these conditions arise when one is either married or widowed. This trend was seen both in NFHS-4 and NFHS-5. Usage of cigarettes, khaini, and alcohol when compared to non-smokers and non-drinkers were strong predictors for these conditions. The chances of having any of these three conditions increased drastically when patients had self-reported heart disease, asthma, thyroid, or cancer.

Logistic regression analysis was applied to adjust age with various variables for calculating the adjusted Odds Ratio for the occurrence of DM, HTN and their co-presence. (Tables 5) As noted, education seems to have an odds ratio showing more education may be less related with these diseases, e.g. Both odds were 0.43(95%CI 0.39-0.47), however when adjusted for age, education followed a similar trend of greater risks getting both HTN and DM aOR 1.40 (95%CI 1.29-1.53).

State-wise prevalence heat map in India according to overall prevalence.

A state-wise heat map representing existing prevalence and earlier recorded prevalence from NFHS-5 and NFHS-4 across the states of all the diseases separately and their co-presence is shown in Supplemental figures 1,2 and 3. The difference in the prevalence of HTN across the states was statistically significant as per unpaired T test with $p<.05$ while for DM and presence of both HTN and Type 2 DM, this difference was not of statistical significance.As per NFHS-5 data , Sikkim state had the highest total prevalence of HTN with 32.21% which is an increase of 7.90% from NFHS-4, while the states of Puducherry and Kerala were having the highest prevalence percentage of DM at 6.20% and 6.60% respectively in NFHS-5 . Kerala had an increase of 0.5% whereas Puducherry had an increase of 0.70% with respect to NFHS-4. As per NFHS-5 highest prevalence for presence of both HTN and DM was observed in Chandigarh >3.0%. Chandigarh noted an increase of 2.10% from NFHS-4.

Various co-morbid conditions were reported by the participants, we plotted a bar graph of these conditions. In NFHS-5 chronic kidney disease was introduced among the questions

Self-Reported thyroid disease and self-reported heart diseases were the major comorbid conditions reported by respondents while the absolute numbers of such conditions were markedly more in hypertensives. Percentage-wise self-reported asthma increased both in hypertensive females and males are shown in supplementary figure 1 and 2. The number of such diseases reported rose in NFHS-5 as compared to NFHS-4. Supplementary table 3 shows the multivariate logistic regression applied on self-reported disorders in NFHS-4 and 5. In self-reported cancer, the odds were seen to be as high as 37 times to 20 times for predicting the presence of DM, the trend of cancer being a strong predictor was consistent in NFHS-4 and NFHS-5.

Table 1 : Study Participant details having only DM as per NFHS 4 and NFHS 5 survey

	NFHS 4		NFHS 5	
	Diabetes N= 16934(2.2)		Diabetes N=23896(3.1)	
	Female	Male	Female	Male
**Age**				
15-19	429(0.4)	103(0.6)	1031(0.9)	176(1.1)
20-24	644(0.5)	120(0.8)	1260(1.1)	179(1.4)
25-29	983(0.9)	201(1.3)	1778(1.6)	233(1.8)
30-34	1478(1.6)	311(2.2)	2219(2.3)	346(2.8)
35-39	2261(2.6)	460(3.5)	3371(3.7)	556(4.7)
40-44	3256(4.4)	606(5.3)	4240(5.6)	621(6.3)
45-49	4506(6.5)	767(7.2)	6077(7.8)	883(8.9)
=>50	0	809(9.9)	0	926(11.9)
Total	13557(2.0)	3377(3.2)	19976(2.9)	3920(4.20)
**Wealth Index**				
Poorest	1327(1.0)	308(1.7)	2817(2.2)	467(2.7)
Poorer	1859(1.3)	466(2.1)	3718(2.6)	694(3.6)
Middle	2533(1.8)	645(2.8)	4077(2.9)	800(4.2)
Richer	3640(2.7)	874(3.9)	4396(3.7)	862(4.6)
Richest	4198(3.3)	1084(5.0)	4968(3.7)	1097(5.9)
**Level of Education**				
None	4123(2.2)	389(2.7)	5391(3.40)	452(4.0)
Incomplete Education	0	0	2897(3.60)	488(4.50)
Incomplete Primary	980(2.5)	230(3.3)	–	-
Primary	1075(2.3)	207(3.0)	–	-
Incomplete Secondary	5084(1.9)	1474(2.9)	9144(2.80)	2183(4.30)
Complete Secondary	962(1.6)	402(3.3)	355(1.50)	96(2.50)
Higher	1333(1.7)	675(4.1)	2189(2.30)	701(4.3)
**BMI**				
<18.5	1057(0.7)	277(1.3)	1500(1.3)	275(2.0)
18.5-22.9	3462(1.1)	1043(2.1)	5874(1.9)	1113(2.7)

23-24.9	1938(2.3)	652(2.9)	3092(3.2)	779(4.8)
25.0-27.4	2434(4.0)	640(5.7)	3310(4.6)	786(6.6)
27.5-29.9	1802(5.7)	392(8.3)	2303(6.1)	433(8.5)
>30.	2428(8.6)	349(12.2)	3261(9.1)	399(12.0)
**Residence**				
Urban	5914(3.0)	1371(4.1)	6685(4.0)	1341(5.7)
Rural	7643(1.6)	2006(2.7)	13291(2.6)	2579(3.7)
**Marital Status (Currently in Union)**				
Never	846(0.5)	377(1.0)	1777(1.1)	512(1.5)
Married	11687(2.4)	2941(4.4)	16737(3.5)	3330(5.7)
Widowed	810(4.2)	37(4.6)	1157(5.6)	44(6.5)
Divorced	71(2.4)	8(2.4)	102(3.9)	15(4.4)
No Longer Living Together with Partner	143(3.2)	14(3.4)	203(4.0)	19(4.6)
**Tobacco use**				
Cigarette	72(3.3)	618(3.8)	58(4.5)	666(5.0)
**Number of Cigarettes in last 24 hours**				
1-4	45(3.0)	257(3.1)	49(4.7)	408(4.7)
5-10	15(3.9)	123(4.3)	5(3.45)	174(6.1)
>10	12(4.5)	135(4.7)	4(4.2)	84(4.9)
Chewing Tobacco	264(1.9)	436(2.9)	146(3.0)	94(4.55)
No smoke	13485(2.0)	2759(3.0)	19918(2.9)	3254(4.1)
**Alcohol Use**				
Drinks alcohol	322(2.0)	1262(3.8)	372(2.9)	1217(5.0)
Not drinks alcohol	13235(2.0)	2115(2.9)	19604(2.90	2703(3.9)
**Waist Hip Ratio NFHS 5**				
≤1	-	-	18852(2.8)	3217(3.6)
>1	-	-	177(4.2)	84(10.6)

- Percentages in parentheses
- \- Data Not available

Table 2 Study Participant details having only HTN as per NFHS 4 and NFHS 5 survey

	NFHS 4		NFHS 5	
	Hypertension N= 101790(14.0)		Hypertension N=110245(14.5)	
	Female	Male	Female	Male
**Age**				
15-19	4469(3.9)	855(4.9)	4517(3.9)	741(5.2)
20-24	6522(5.99)	1425(9.5)	6162(5.6)	1317(10.7)
25-29	8949(8.7)	2055(14.2)	9098(8.3)	1828(15.1)
30-34	11490(13.3)	2545(19.4)	11729(12.5)	2443(21.8)
35-39	15007(18.6)	2976(23.9)	16502(18.1)	2911(26.2)
40-44	16714(24.4)	3045(28.3)	18889(24.9)	3028(32.3)
45-49	19736(30.3)	3254(32.5)	24543(31.2)	3438(36.2)
=>50	0	2748(35.7)	0	3120(41.4)
Total	82887(13.2)	18903(18.7)	91440(13.5)	18826(21.6)
**Wealth Index**				

Poorest	13695(11.1)	2338(13.8)	16539(12.7)	3172(19.2)
Poorer	16583(12.2)	3258(15.4)	18655(13.1)	3580(19.7)
Middle	17090(13.0)	4014(18.3)	18584(13.4)	3821(21.3)
Richer	17901(14.6)	4466(21.6)	18395(13.7)	3959(22.5)
Richest	17618(15.3)	4827(23.9)	19267(14.8)	4294(25.1)
**Level of Education**				
None	29831(16.6)	2706(19.9)	29856(19.1)	2598(24.40)
Incomplete Education	0	0	13632(17.10)	2530(24.70)
Incomplete Primary	6140(16.6)	1410(21.5)	-	-
Primary	6200(14.4)	1213(18.7)	-	-
Incomplete Secondary	28219(11.5)	8060(17.0)	37650(11.70)	9971(20.80)
Complete Secondary	5450(10.1)	2149(18.7)	1574(6.70)	478(13.20)
Higher	7047(10.1)	3365(21.7)	8707(9.40)	3249(21.70)
**BMI**				
<18.5	10037(7.1)	1685(8.5)	8107(7.0)	1220(9.4)
18.5-22.9	27954(9.8)	6922(14.4)	29410(9.7)	6211(16.2)
23-24.9	12534(16.4)	3794(24.5)	14813(15.7)	3768(25.1)
25.0-27.4	12616(22.8)	3416(32.8)	14774(20.8)	3662(33.1)
27.5-29.9	8148(28.2)	1833(41.6)	9852(26.4)	1971(41.2)
>30.	9124(35.8)	1180(44.2)	11550(32.7)	1421(44.6)
**Residence**				
Urban	25900(14.4)	6726(21.4)	24342(14.8)	5450(24.6)
Rural	56987(12.7)	12177(17.5)	67098(13.1)	13376(20.5)
**Marital Status (Currently in Union)**				
Never	8294(5.3)	3354(9.2)	8777(5.2)	3224(10.4)
Married	69149(15.5)	15172(24.0)	75951(15.9)	15189(27.7)
Widowed	4330(23.8)	209(27.5)	5400(26.0)	223(33.7)
Divorced	490(17.7)	82(25.6)	444(17.0)	77(24.1)
No Longer Living Together with Partner	624(14.7)	86(21.7)	868(16.8)	113(27.6)
**Tobacco use**				
Cigarette	343(16.8)	3204(20.8)	242(18.5)	3007(24.4)
**Number of Cigarettes in last 24 hours**				
1-4	220(16.2)	1529(19.3)	192(18.2)	1922(23.6)
5-10	66(17.6)	572(21.8)	32(20.6)	672(26.3)
>10	52(20.6)	657(24.3)	18(18.8)	413(25.60)
Chewing Tobacco	2453(18.2)	2943(20.80	823(16.6)	510(25.6)
No smoke	82544(13.2)	15699(18.3)	91198(13.5)	15189(21.1)
**Alcohol Use**				
Drinks alcohol	3226(22.1)	7509(23.9)	2988(23.1)	6760(29.2)
Not drinks	79661(13.0)	11394(16.4)	88452(13.4)	12066(18.8)

alcohol				
**Waist Hip Ratio NFHS 5**				
<=1	-	-	88013(13.4)	16647(20.2)
>1	-	-	660(15.9)	271(36.0)

Table 3: Study Participant details having both HTN and DM as per NFHS 4 and NFHS 5

	NFHS 4		NFHS 5	
	Both HTN & DM N=5936(0.8)		Both HTN & DM N=8235(1.1)	
	Female	Male	Female	Male
**Age**				
15-19	26(0.0)	5(0.9)	99(1.5)	15(0.1)
20-24	61(0.1)	15(0.1)	140(2.1)	35(0.3)
25-29	162(0.2)	41(0.3)	286(4.3)	34(0.3)
30-34	360(0.4)	85(0.7)	514(7.7)	101(0.9)
35-39	750(1.0)	158(1.3)	1128(16.9)	192(1.8)
40-44	1300(1.9)	246(2.3)	1691(25.3)	269(3.0)
45-49	1978(3.1)	357(3.6)	2824(42.3)	429(4.7)
=>50	0	392(5.2)	0	478(6.7)
Total	4637(0.8)	1299(1.3)	6682(1.0)	1553(1.8)
**Wealth Index**				
Poorest	316(0.3)	84(0.5)	778(0.6)	141(0.9)
Poorer	547(0.4)	138(0.7)	1169(0.9)	253(1.4)
Middle	822(0.6)	209(1.0)	1376(1.0)	302(1.7)
Richer	1356(1.1)	394(1.9)	1506(1.2)	357(2.1)
Richest	1596(1.4)	474(2.4)	1853(1.5)	500(3.0)
**Level of Education**				
None	1515(0.9)	138(1.0)	1963(1.30)	173(1.70)
Incomplete Education	0	0	1132(1.50)	211(2.10)
Incomplete Primary	357(1.0)	98(1.5)	-	-
Primary	406(1.0)	86(1.3)	-	-
Incomplete Secondary	1677(0.7)	559(1.2)	2940(0.90)	852(1.80)
Complete Secondary	298(0.6)	137(1.2)	82(.40)	28(0.80)
Higher	384(0.6)	281(1.8)	565(0.60)	289(2.00)
**BMI**				
<18.5	162(0.1)	41(0.2)	229(0.2)	53(0.4)
18.5-22.9	773(0.3)	302(0.6)	1274(0.4)	333(0.9)
23-24.9	657(09)	228(1.5)	975(1.1)	313(2.1)
25.0-27.4	912(1.7)	311(3.0)	1263(1.8)	362(3.4)
27.5-29.9	794(2.8)	226(5.2)	1041(2.9)	218(4.8)
>30.	1205(4.8)	184(7.0)	1670(4.9)	218(7.25)
**Residence**				

Urban	6726(21.4)	2225(1.3)	2515(1.6)	607(2.8)
Rural	12177(17.5)	2412(0.5)	4167(0.8)	946(1.5)
**Marital Status (Currently in Union)**				
Never	137(0.1)	61(0.2)	254(0.2)	87(0.3)
Married	4092(0.9)	1214(1.9)	5811(1.3)	1430(2.7)
Widowed	350(2.0)	16(2.1)	510(2.6)	25(4.0)
Divorced	24(0.9)	3(`1.0)	39(1.5)	6(1.9)
No Longer Living Together with Partner	34(0.8)	5(1.3)	68(1.4)	5(1.3)
**Tobacco use**				
Cigarette	24(1.3)	233(1.5)	22(1.8)	256(2.2)
**Number of Cigarettes in last 24 hours**				
1-4	13(1.0)	103(1.3)	19(1.9)	160(2.0)
5-10	6(1.7)	40(1.6)	1(0.7)	63(2.6)
>10	5(2.1)	49(1.9)	2(2.2)	33(2.2)
Chewing Tobacco	103(0.80	171(1.2)	57(1.2)	42(2.20)
No smoke	4613(0.8)	1066(1.3)	6660(1.0)	1297(1.8)
**Alcohol Use**				
Drinks alcohol	102(0.7)	530(1.7)	131(1.1)	546(2.5)
Not drinks alcohol	4535(0.8)	769(1.1)	6551(1.0)	1007(1.6)
**Waist Hip Ratio NFHS-5**				
<=1	-	-	6153(1.0)	1167(1.5)
>1	-	-	70(2.0)	51(7.1)

Table 4: Multivariate Regression analysis for obtaining odds of various correlates with the occurrence of either DM, HTN or both concurrently as per NFHS-4 and NFHS-5

	Diabetes Values as OR (95CI) *denotes p value<.05		Hypertension Values as OR (95CI) *denotes p value<.05		Both (Values as OR (95CI) *denotes p value<.05	
	NFHS 4	NFHS 5	NFHS 4	NFHS 5	NFHS 4	NFHS 5
**Age Groups**						
15-19	1	1	1	1	1	1
20-24	1.47* (1.32-1.65)	1.23* (1.14-1.33*	1.60* (1.55-1.66)	1.53* (1.47-1.58)	2.57* (1.69 - 3.91)	1.61* (1.27-2.04)
25-29	2.45* (2.21-2.71)	1.73* (1.61-1.86)	2.48* (2.4-2.56)	2.32* (2.24-2.40)	7.44* (5.10-10.87)	2.98* (2.41-3.69)
30-34	4.38* (3.97-	2.59* (2.42-2.78)	3.92* (3.79-	3.69* (3.56-3.80)	19.26* (13.38-	6.71* (5.50-8.20)

	4.82)		4.05)		27.73)	
35-39	7.22* (6.58-7.93)	4.17* (3.91-4.45)	5.72* (5.54-5.91)	5.52* (5.34-5.7)	42.36* (29.61-60.6)	14.98* (12.37-18.15)
40-44	12.33* (11.25-13.5)	6.38* (5.98-6.80)	7.92* (7.76-8.17)	8.17* (7.92-8.43)	85.53* (59.93-122.0)	27.20* (22.52-32.86)
45-49	18.23* (16.67-19.9)	9.11* (8.57-9.69)	10.52* (10.2-10.85)	10.95* (10.62-11.3)	138.40* (97.09-197.2)	44.43* (36.85-53.57)
>50	28.37* (25.37-31.7)	14.36* (13.14-15.7)	13.26* (12.56-14.0)	16.65* (15.78-17.6)	230.61* (159.6-332.7)	79.55* (64.76-97.73)
**BMI**						
<18	1	1	1	1	1	1
18.5-22.9	1.62* (1.52-1.72)	1.49* (1.42-1.57)	1.49* (1.46-1.52)	1.49* (1.46-1.53)	2.58* (2.22-2.99)	2.15* (1.90-2.43)
23-24.9	3.36* (3.14-3.59)	2.60* (2.46-2.75)	2.75* (2.68-2.82)	2.61* (2.54-2.68)	7.75* (6.65-9.03)	5.42* (4.78-6.15)
25.0-27.4	5.62* (5.26-5.99)	3.73* (3.53-3.95)	4.09* (3.99-4.20)	3.71* (3.62-3.81)	15.08* (13.01-17.5)	9.27* (8.19-10.5)
27.9-29.9	8.07* (7.53-8.65)	4.93* (4.65-5.24)	5.46* (5.29-5.62)	4.99* (4.84-5.14)	25.23* (21.69-29.35)	14.15* (12.46-16.07)
30 or above.	12.39* (11.6-13.24)	7.46* (7.04-7.90)	7.36* (7.13-7.58)	6.49* (6.30-6.69)	41.43* (35.74-48.03)	23.76* (21.01-26.87)
**Waist /Hip Ratio for NFHS-5**						
<1	-	1	-	1	-	1
>1	-	1.82* (1.60-2.06)	-	1.42* (1.33-1.53)	-	2.78* (2.33-3.31)
**Residence**						
Rural	0.54* (0.52-0.55)	0.56* (0.47-0.66)	0.84* (0.83-0.85)	0.85* (0.84-0.86)	0.44* (0.42-0.47)	0.52* (0.50-0.54)
Urban	1	1	1	1	1	1
**Education**						
None	1	1	1	1	1	1
Incomple	-	1.07*	-	0.98	-	1.06

te Education		(1.03-1.12)		(0.85-1.14)		(0.98-1.14)
Primary incomplete	1.19* (1.12-1.27)	-	1.03 (1.01-1.06)	-	1.23* (1.11-1.37)	-
Primary complete	1.10* (1.04-1.17)	-	0.87* (0.84-0.89)	-	1.16 (1.05-1.28)	-
Secondary incomplete	0.94* (0.91-0.98)	0.86* (0.83-0.89)	0.69* (0.68-0.70)	0.86* (0.76-0.97)	0.89* (0.83-0.95)	0.67* (0.64-0.71)
Secondary Complete	0.86* (0.81-0.92)	0.46* (0.42-0.51)	0.64* (0.63-0.66)	0.59* (0.44-0.79)	0.77* (0.69-0.85*	0.25* (0.20-0.31)
Higher	0.98 (0.92-1.03)	0.74* (0.71-0.78)	0.68* (0.66-0.69)	0.81* (0.69-0.94)	0.94* (0.82-0.99)	0.43* (0.39-0.47)
**Marital Status**						
Never	1	1	1	1	1	1
Married	4.5* (4.24-4.77)	3.36* (3.22-3.51)	3.07* (3.01-3.13)	2.39* (2.16-2.66)	10.22* (8.86-11.78)	5.67* (3.54-9.08)
Widowed	7.17* (6.56-7.84)	5.22* (4.86-5.60)	4.89* (4.71-5.08)	3.31* (2.42-4.54)	19.35* (16.27-23.02)	11.04* (4.89-24.93)
Divorced	4.09* (3.24-5.14)	3.62* (2.99-4.37)	3.53* (3.21-3.87)	1.92* (1.23-3.01)	8.77* (5.86-13.14)	1.58 (0.20-12.00)
No longer living together	5.40* (4.56-6.39)	3.65* (3.17-4.20)	2.81* (2.59-3.05)	2.27* (1.41-3.66)	8.32* (5.89-11.47)	5.12* (1.16-22.50)
**Wealth Quintile**						
Poorest	1	1	1	1	1	1
Poorer	1.25* (1.17-1.33)	1.23* (1.17-1.28)	1.19* (1.09-1.14)	1.06 (0.94-1.20)	1.53* (1.35-1.73)	1.64* (1.07-2.51)
Middle	1.73* (1.63-1.84)	1.39* (1.22-1.45)	1.23* (1.20-1.26)	1.25* (1.10-1.42)	2.36* (2.10-2.65)	2.04* (1.34-3.12)
Richer	2.64* (2.50-	1.55* (1.48-1.62)	1.43* (1.40-	1.33* (1.17-1.51)	4.31* (3.85-4.80)	1.85* (1.18-2.88)

	2.80)		1.46)			
Richest	3.29* (3.11-3.48)	1.83* (1.75-1.91)	1.53* (1.50-1.57)	1.48* (1.29-1.69)	5.38* (4.83-5.99)	3.51* (2.32-5.30)
**Tobacco Use**						
Cigarette	1.76* (1.63-1.90)	1.65* (1.53-1.78)	1.59* (1.53-1.65)	1.16* (1.04-1.17)	1.88* (1.65-2.13)	1.04 (.88-1.23)
Khaini	1.13* (1.05-1.22)	1.27* (0.99-1.28)	1.52* (1.47-1.57)	1.17* (1.10-1.25)	1.26* (1.11-1.42)	1.18 (0.97-1.45)
No smoke	1	1	1	1	1	1
**Alcohol Use**						
Drinks alcohol	1.53* (1.45-1.61)	1.44* (1.36-1.51)	1.97* (1.93-2.02)	2.29* (2.23-2.34)	1.79* (1.65-1.95)	1.83* (1.69-1.98)
Not drinks alcohol	1	1	1	1	1	1

Table 5: Multivariate Logistic regression analysis for various variables with adjusted OR like age, BMI and marital status with 95 CI for having HTN , Diabetes and Both of NFHS-4 and NFHS -5.

	NFHS 4	NFHS 5	NFHS 4	NFHS 5	NFHS 4	NFHS 5
	Diabetes	Diabetes	Hypertension	Hypertension	Both	Both
	aOR (95% CI)	aOR (95% CI)	aOR (95% CI)	aOR (95% CI)	aOR (95% CI)	aOR (95% CI)
**Male/Female**						
15-19	1	1	1	1	1	1
20-24	1.32* (1.17-1.49)	1.07 (0.98-1.16)	1.6* (1.54-1.66)	1.43* (1.37-1.49)	2.17* (1.41-3.35)	1.20 (0.93-1.55)
25-29	1.96* (1.73-2.21)	1.07* (1.20-1.43)	2.29* (2.21-2.39)	2.02* (1.94-2.11)	5.36* (3.58-8.03)	1.77* (1.38-2.26)

30-34	3.20* (2.84-3.61)	1.83* (1.67-2.00)	3.36* (3.23-3.51)	2.98* (1.94-2.11)	11.89* (8.00-17.69)	3.35* (2.63-4.26)
35-39	5.14* (4.57-5.79)	2.86* (2.62-3.13)	4.75* (4.56-4.96)	4.32* (4.14-4.50)	24.55* (16.57-36.37)	6.83* (5.40-8.64)
40-44	8.66* (7.70-9.75)	4.32* (3.96-4.72)	6.43* (6.17-6.71)	6.25* (6.00-6.52)	47.42* (32.06-70.15)	11.93* (9.45-15.05)
45-49	13.07* (11.63-14.7)	6.33* (5.81-6.90)	8.50* (8.15-8.87)	8.40* (8.06-8.76)	50.54* (42.03-63.72)	19.52* (15.49-24.60)
>50	20.94* (18.30-23.96	10.05* (9.02-11.2)	11.53* (10.85-12.27)	12.76* (11.98-13.59)	65.32* (52.26-72.63)	35.37* (27.47-45.53)
**BMI**						
<18	1.00	1.00	1.00	1.00	1.00	1.00
18.5-22.9	1.30* (1.22-1.38)	1.21* (1.15-1.28)	1.28* (1.26-	1.16* (1.22-1.19)	1.93* (1.66-2.25)	1.53* (1.35-1.74)
23.0-24.9	1.99* (1.86-2.13)	1.66* (1.57-1.76)	1.92* (1.87-	1.64* (1.59-1.68)	3.96* (3.39-4.62)	2.72* (2.39-3.10)
25.0-27.4	2.95* (2.76-3.16)	2.19* (2.06-2.32)	2.64* (2.57-	2.16* (2.10-2.22)	6.66* (5.72-7.74)	4.17* (3.67-4.74)
27.5-29.9	3.94* (3.66-4.23)	2.75* (2.58-2.93)	3.36* (3.25-	2.75* (2.66-2.84)	10.21* (8.75-11.90)	5.95* (5.21_6.79)
>30	5.77* (5.39-6.19)	4.06* (3.82-4.32)	4.40* (4.26-	3.55* (3.44-3.67)	15.88* (13.66-18.47)	9.81* (8.63-11.15)
**Marital Status**						

Never	1.00	1.00	1.00	1.00	1.00	1.00
Married	1.01 (0.94-1.10)	1.09 (0.94-1.26)	0.85 (0.82-	0.91 (0.88-0.94)	0.86 (0.73-1.02)	1.11* (1.05-1.19)
Widowed	1.06 (0.95-1.18)	1.23* (1.03-1.46)	0.91 (0.86-	1.03 (0.99-1.08)	0.99 (0.82-1.21)	1.16* (1.06-1.27)
Divorced	0.95 (0.75-1.21)	1.24 (0.87-1.75)	0.98 (0.88-	0.94 (0.84-1.04)	0.81 (0.53-1.22)	1.20 (0.98-1.47)
No longer living together	1.27* (1.05-1.52)	1.07 (0.80-1.43)	0.78 (0.71-	0.95 (0.88-1.03)	0.77 (0.53-1.10)	1.19* (1.02-1.39)
**Education**						
No Education	1.00	1.00	1.00	1.00	1.00	1.00
Incomplete Primary	1.31* (1.22-1.40)	1.30* (1.24-1.35)	1.14* (1.11-	1.11* (1.08-1.13)	1.33* (1.19-1.48)	1.47* (1.37-1.58)
complete Primary	1.29* (1.21-1.38)	-	1.01 (0.98-	-	1.32* (1.19-1.46)	-
Incomplete Secondary	1.50* (1.44-1.57)	1.50* (1.45-1.55)	1.05* (1.03-	1.11* (1.09-1.13)	1.42* (1.33-1.52)	1.62* (1.53-1.71)
complete Secondary	1.46* (1.37-1.56)	1.37* (1.23-1.51)	0.98 (0.95-	1.09* (1.04-1.15)	1.31* (1.17-1.47)	1.42* (1.16-1.72)
Higher	1.47* (1.39-1.56)	1.37* (1.30-1.44)	0.90 (0.87-	1.00 (0.97-1.02)	1.34* (1.22-1.48)	1.40* (1.29-1.53)

Discussion

To our knowledge in our study is the first study to compare the findings of NFHS-4 and NFHS-5 for the whole country data. We have attempted to analyze and compare the findings of NFHS-4 and NFHS-5 data bases looking for overall prevalence of HTN, DM and concurrent presence of both HTN and DM together. More than 16 lakhs data samples were analyzed for finding the various correlates responsible for odds of these diseases across the country. The present study has mapped state wise prevalence of the above mentioned morbidities. Results of the study shows the trend of the disease in relationship with predictors of disease such as gender, age, residence, literacy, wealth index, BMI, waist hip ratio etc to ascertain their relationship and role.

1.Summary of Key findings of the study

We found that the prevalence of HTN, DM, and the concurrent presence of both has increased in India. This is the latest comparison of both country-wide exhaustive data sets in the Indian context. The increase in prevalence is more marked in the age group of 30-50. This suggests that all cases who are coming for DM or HTN should be screened for both, either in a diabetic clinic or a clinic for heart care.

2. Comparison of Diabetes in the database of NFHS-4 and NFHS-5.

The prevalence of DM was 2.2 and 3.1 as per NFHS-4 and NFHS-5 data respectively showing an increasing trend. In NFHS-5 the prevalence and risk of DM were seen more in males, age group > 50 years, richest wealth index, people having incomplete education, BMI > 30 kg/m2, urban residents, widowed, smokers, alcoholics and WHR > 1.The highest prevalence of DM across the states was seen in Puducherry (6.6). We noted an increasing trend regarding odds of having DM as the individual aged. The adjusted odds for age with other factors also had similar trend. Other studies about NFHS-5 also had similarity regarding the prevalence of DM in old aged individuals.[14] Such trends of overall prevalence in these states were seen earlier in NFHS-4 data analysis by Geldsetzer, Pascal et al earlier.[8] As noted in our study we noted higher odds of having DM for people living in urban areas these odds were similar even after adjustment. Large studies done in Indian context have reported similar findings.[15] Marital status and association of DM was seen to be statistically associated in cases of separated individuals, the trends in NFHS-4 and NFHS-5 were statistically significant when odds were calculated. The factors have been highlighted by previous study done in India.[15] Possible explanation has generally been given as lack of care and stress of living alone, and sleep deprivation. Other studies have established possible mechanism for this strong association.[16] Odds of having DM was also related with educational status and it was higher in higher study groups. Earlier studies have reported association of DM in Iran with education in Asian context.[17] Possible explanation has been nature of employment which is generally associated with higher education, higher income and sedentary life style. Similarly higher wealth index, use of tobacco and consumption of alcohol were seen to be strong risk factors for occurrence of DM. Studies in the past have highlighted the mechanism by which alcohol tends to increase the sugar levels and thus by worsening the DM.[17]

3. Comparison of HTN in the database of NFHS-4 and NFHS-5.

HTN prevalence was 14 and 14.5 as per NFHS-4 and NFHS-5 data respectively. NFHS-5 data reveals that the prevalence and risk of HTN has increased in males, age group >50 years,

richest wealth index, people having no education, BMI > 30 kg/m2, urban residents, widowed, smokers, alcoholics and WHR > 1. Prevalence for HTN has increased by 7 in Sikkim while in Nagaland it has decreased when compared with NFHS-4 data.

Other authors like Vennu et al. have raised this issue in their NFHS-4 and other analysis involving large country data set. [18,19] In our study overall prevalence of HTN in NFHS-5 was 14.5 while for male it was 21.6 and females it was 13.5. This prevalence was more markedly increased in male with a rise of approx. 3. Of note in the various states about the prevalence of HTN in Kerala as it has been noted that despite the epidemiological transition in NFHS-4 the state had a lower prevalence as compared to other states.[20] The percentage of prevalence has risen from 11.5 to 13.5 in NFHS-5 possible explanations could be the large number of female participants and age less than 60 years. As noted by Gosh et al hypertension in India is no longer a disease afflicting mostly the rich, but now the poor are also having a higher prevalence of HTN.[20] Percentages of prevalence in the poor have increased in NFHS-4 as compared to NFHS-5. We noted educational attainment to be inversely proportional to the prevalence of HTN. This was also noted by Gosh et al in their NFHS-4 analysis and in NFHS-5 trend is same. Persons residing in rural areas had lesser chances of getting HTN as the odds were lesser with statistical significance. This was also seen in NFHS-4 data by Gosh et al.[20] Obesity and alcohol have strong relationship with HTN as seen in both NFHS-4 and NFHS-5 and this similar relationship of these variables in Indian large population studies have been reported earlier by Bhansali et al.[21] We noted strong association of smokers and tobacco users with HTN prevalence. Ghosh et al had little different finding then us in their NFHS-4 data analysis. However, our findings are consistent with available evidence from various Indian studies which has attributed cigarette smoking , tobacco use and obesity as the top contributors apart from age for HTN.[22]

4. Comparison of concurrent presence of both DM and HTN together in the database of NFHS-4 and NFHS-5.

Bischops, Anne C et al in their large study found overall prevalence of HTN to be 22.1 while that for raised blood sugar was 6.4 and prevalence of having concurrently raised blood sugar and HTN was 1.5.[11] In NFHS 5 we found the concurrent occurrence of DM and HTN to be 1.1. Tripathi et al. in their study found an estimated prevalence of 4.5 which was way higher than our findings.[23] We can say that our findings are close enough when considered with the findings of Bischops et al.[11] We noted higher concurrent prevalence of DM and HTN in older age, high BMI group individuals , more in individuals having higher waist hip ration >1 , having higher wealth index and having higher education. Corsi et al in their analysis of NFHS 4 had similar results.[24] Large data set studies of national representative population in similar settings have revealed the same predictors regarding the concurrent presence of HTN and DM.[25]

Our findings about the prevalence of DM, HTN and presence of both had interesting findings state wise, as we noted relatively higher prevalence of HTN in the north easter states of Sikkim (Highest in India), Arunachal Pradesh and Nagaland in both NFHS 4 and 5.

Older age groups were key vulnerable groups which were seen to have more prevalence of either DM, HTN, or concurrent DM and HTN.

5. Pathological basis for correlation between co presence of HTN along with DM and its ramifications.

Recent studies have shown that hypertension can lead to diabetes in the same individual. Song et al [26] in their cohort study of 3017 Japanese participants were unique in forecasting that long-term blood pressure/HTN is a risk factor for type 2 diabetes. Tanaka et al [27] have subsequently explained using other large cohort studies how HTN uncontrolled blood pressure promotes insulin resistance through known and unknown pathways to cause DM. This mechanism is unclear, but alarm bells must be rung to emphasise this relation as we uncover it.

If we don't take timely measures to monitor the prevalence of DM and HTN across different states in India, we risk overlooking cases and ending up with a larger number of individuals requiring treatment for both conditions, which could lead to a heavier burden on our economy. Given India's vast size, it is crucial that we focus on this issue to prevent such outcomes.

Strengths of Study

The study's strengths included a large, nationally representative survey dataset and a systematic analysis procedure. Our findings apply to more regions. Second, a comprehensive sample analysis design was used to consider sampling units and weighting. In addition, the study identified the high-risk group and the Indian states with the lowest blood pressure and glucose screening rates. Third We compared three-year-old country data bases. Trends from such a vast data set can forecast future events in India. We followed the definition of HTN as per the ESC guidelines for hypertension and its various operational definition[28] .

Conclusion

As can be seen in the comparative trend the overall prevalence of chronic diseases in Indian states and population has risen over the years. This trend follows an upward slope. The worrisome part is the rise in individuals having the concurrent presence of DM and HTN. This mandates more research for such individuals and more extensive screening for looking out for such individuals. Cross-referral form cardiac screening /treatment clinics for DM screening and vice versa should be the norm rather than necessity. Resource allocation for early diagnosis of DM, HTN and their concurrent presence can be done on the basis of prevalence in various states. Public needs to be made aware about the various threats these two major non communicable diseases pose to them.

Limitations of the study

Since only data from females aged 15-50 was available, this study cannot reflect the entire population. NFHS 4 and 5 prioritised maternal and child health, hence male samples were

smaller than female samples. Data from >2 lakh males is sufficient for country-specific estimates. We used blood glucose levels from the data set and cannot tell if the patient had type 2 or type 1 DM. NFHS-4 survey data lacks waist hip ratio data.

Acknowledgment: The authors would like to acknowledge and thank Resource Center for Tobacco control in India (RCTC) established under the Postgraduate Institute of Medical Education and Research (PGIMER), Chandigarh for providing technical support towards writing the manuscript. We are also grateful to Global Health Advocacy Incubator (GHAI) for supporting the study and Demographic and Health Surveys (DHS) Program for providing the data set (survey ref no. 155509 downloaded on June 3, 2022) which helped in the development

of the manuscript.

Contribution

Role of various authors were as following – RKR - Conceptualisation, Data Curation, writing original draft, formal analysis . RRJ - Review. RS- Data curation, Validation,Review , Conceptualisation. DK- Review and Methodology. RJ- Review. UP- Resources and Software. JBP -Data curation and analysis. SSK- Review, SG Conceptualisation, Investigation, Methods, Project Administration, review and Editing. *The final editing was done by all the authors. Lastly, all authors read and approved the final manuscript for publication.*

Conflict of interest*:* None declared.

Data sharing statement: All deidentified data are publicly available and can be accessed through The DHS Program,https://dhsprogram.com/data/.

Funding-*None*

References

1 Heald AH, Stedman M, Davies M, *et al.* Estimating life years lost to diabetes: outcomes from analysis of National Diabetes Audit and Office of National Statistics data. *Cardiovasc Endocrinol Metab* 2020; 9: 183–5.

2 Sun H, Saeedi P, Karuranga S, *et al.* IDF Diabetes Atlas: Global, regional and country-level diabetes prevalence estimates for 2021 and projections for 2045. *Diabetes Res Clin Pract* 2022; 183: 109119.

3 Gakidou E, Afshin A, Abajobir AA, *et al.* Global, regional, and national comparative risk assessment of 84 behavioural, environmental and occupational, and metabolic risks or clusters of risks, 1990–2016: a systematic analysis for the Global Burden of Disease Study 2016. *The Lancet* 2017; 390: 1345–422.

4 Izzo R, de Simone G, Chinali M, *et al.* Insufficient Control of Blood Pressure and Incident Diabetes. *Diabetes Care* 2009; 32: 845–50.

5 Ferrannini E, Cushman WC. Diabetes and hypertension: the bad companions. *The Lancet* 2012; 380: 601–10.

6 Sowers JR. Diabetes Mellitus and Vascular Disease. *Hypertension* 2013; 61: 943–7.

7 Indian Council of Medical Research PHF of I and I for HM and E. GBD India compare data visualization. New Delhi: ICMR, PHFI, and IHME. 2017.

8 Geldsetzer P, Manne-Goehler J, Theilmann M, *et al.* Diabetes and Hypertension in India. *JAMA Intern Med* 2018; 178: 363.

9 Prenissl J, Manne-Goehler J, Jaacks LM, *et al.* Hypertension screening, awareness, treatment, and control in India: A nationally representative cross-sectional study among individuals aged 15 to 49 years. *PLoS Med* 2019; 16: e1002801.

10 OGD PMU Team. Annual Health Survey (AHS) unit level data now available on OGD Platform. District Level Household and Facility Survey. 2016; published online May 19.

11 Bischops AC, Manne-Goehler J, Jaacks LM, *et al.* The prevalence of concurrently raised blood glucose and blood pressure in India. *J Hypertens* 2019; 37: 1822–31.

12 Amarchand R, Kulothungan V, Krishnan A, Mathur P. Hypertension treatment cascade in India: results from National Noncommunicable Disease Monitoring Survey. *J Hum Hypertens* 2022; published online May 5. DOI:10.1038/s41371-022-00692-y.

13 The DHS Program Demographic and Health Survey Team and USAID. The DHS Program . The DHS Program National and Family Health Survey Data files for India 2019-21 DHS V11. 2019. https://dhsprogram.com/methodology/survey/survey-display-541.cfm (accessed Jan 13, 2023).

14 Kshirsagar M, Ashturkar M. Prevalence of lifestyle diseases in Maharashtra: A comparison between NFHS-5 and NFHS-4 surveys. *J Family Med Prim Care* 2022; 11: 2474.

15 Sujata, Thakur R. Unequal burden of equal risk factors of diabetes between different gender in India: a cross-sectional analysis. *Sci Rep* 2021; 11: 22653.

16 Cornelis MC, Chiuve SE, Glymour MM, *et al.* Bachelors, Divorcees, and Widowers: Does Marriage Protect Men from Type 2 Diabetes? *PLoS One* 2014; 9: e106720.

17 Azimi-Nezhad M, Ghayour-Mobarhan M, Parizadeh MR, *et al.* "Prevalence of type 2 diabetes mellitus in Iran and its relationship with gender, urbanisation, education, marital status and occupation." *Singapore Med Journal* 2008; 49: 571–6.

18 Vennu V, Abdulrahman TA, Bindawas SM. The Prevalence of Overweight, Obesity, Hypertension, and Diabetes in India: Analysis of the 2015–2016 National Family Health Survey. *Int J Environ Res Public Health* 2019; 16: 3987.

19 Bansal SK, Goel D, Saxena V, Kandpal SD, Gray WK, Walker RW. The prevalence of hypertension and hypertension risk factors in a rural Indian community: A prospective door-to-door study. *J Cardiovasc Dis Res* 2012; 3: 117–23.

20 Ghosh S, Kumar M. Prevalence and associated risk factors of hypertension among persons aged 15–49 in India: a cross-sectional study. *BMJ Open* 2019; 9: e029714.

21 Bhansali A, Dhandania VK, Deepa M, *et al.* Prevalence of and risk factors for hypertension in urban and rural India: the ICMR–INDIAB study. *J Hum Hypertens* 2015; 29: 204–9.

22 Anchala R, Kannuri NK, Pant H, *et al.* Hypertension in India. *J Hypertens* 2014; 32: 1170–7.

23 Tripathy JP, Thakur JS, Jeet G, Jain S. Prevalence and determinants of comorbid diabetes and hypertension: Evidence from non communicable disease risk factor STEPS survey, India. *Diabetes & Metabolic Syndrome: Clinical Research & Reviews* 2017; 11: S459–65.

24 Corsi DJ, Subramanian S v. Socioeconomic Gradients and Distribution of Diabetes, Hypertension, and Obesity in India. *JAMA Netw Open* 2019; 2: e190411.

25 Duodu PA, Agbadi P, Duah HO, Darkwah E, Nutor JJ. Correlates of blood pressure and blood glucose screenings in Cameroon: insights from the 2018 Demographic and Health Survey. *Int Health* 2022; 14: 201–10.

26. Song Z, He Y, Chiang C, Al-shoaibi A, Saif-Ur-Rahman KM, Mamun MR, et al. Long-term variability and change trend of systolic blood pressure and risk of type 2 diabetes mellitus in middle-aged Japanese: findings of Aichi Workers' Cohort Study. Hypertens Res. 2022. https://doi.org/10.1038/s41440-022-00993-2.

27. Tanaka, A., Node, K. Pathogenic connection between hypertension and type 2 diabetes: how do they mutually affect each other?. *Hypertens Res* **45**, 1840–1842 (2022). https://doi.org/10.1038/s41440-022-01014-y

28.Williams B, Mancia G, Spiering W, et al. 2018 ESC/ESH Guidelines for the management of arterial hypertension [published correction appears in Eur Heart J. 2019 Feb 1;40(5):475]. *Eur Heart J.* 2018;39(33):3021-3104. doi:10.1093/eurheartj/ehy339

 Click here to access/download;Figure;Figure 1.docx

Supplementary figures showing the state wise prevalence of DM , HTN and concurrent presence of both across states in India .

Figure 1

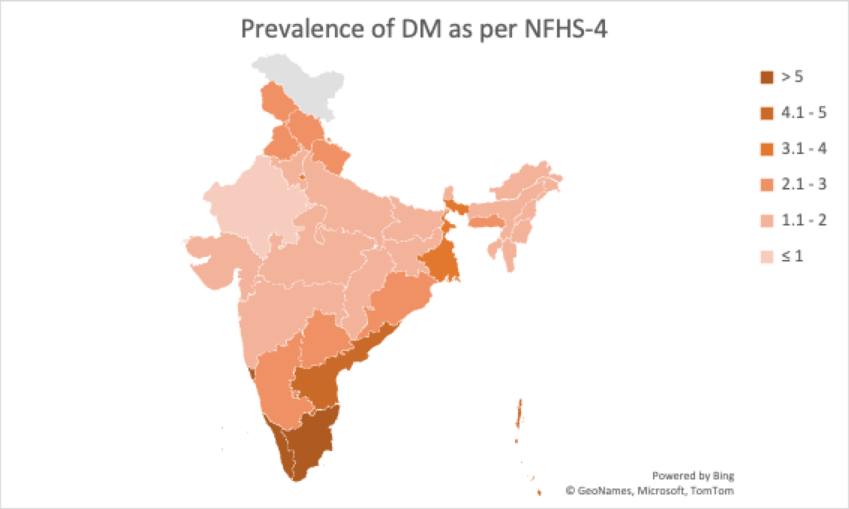

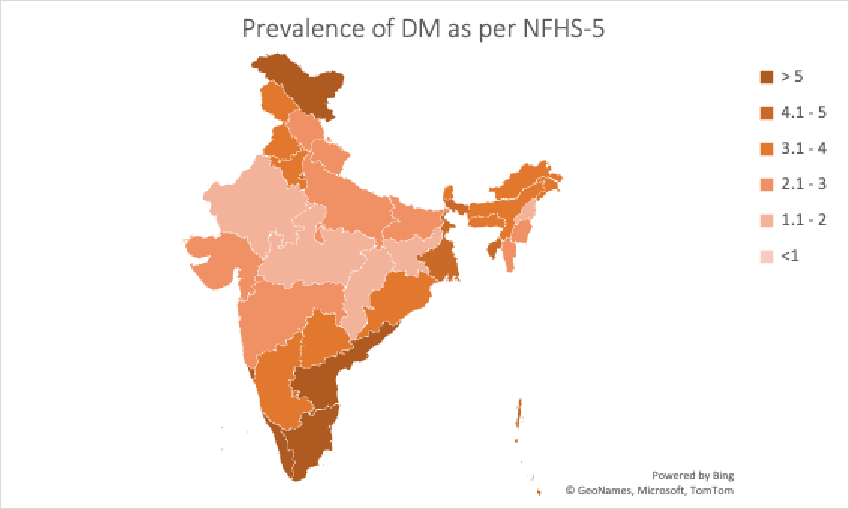

Figure 1. Prevalence of DM (% wise) as per NFHS 4 and 5 in States across India .

Figure 2

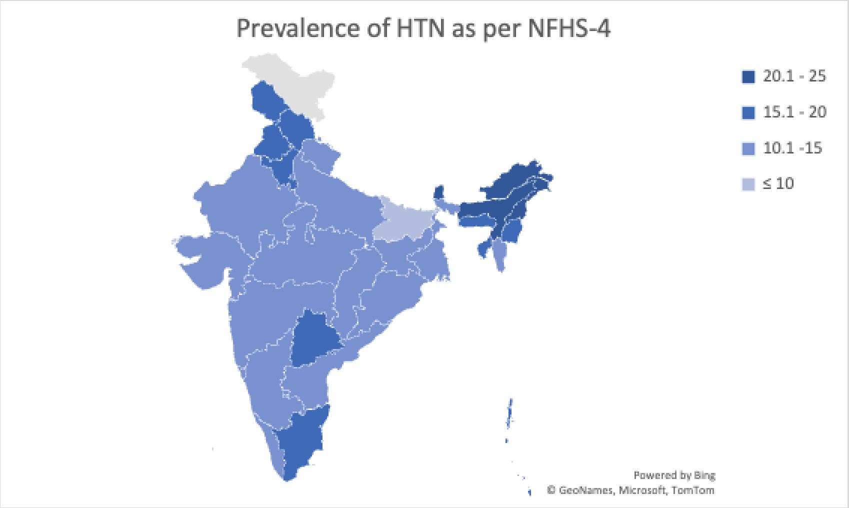

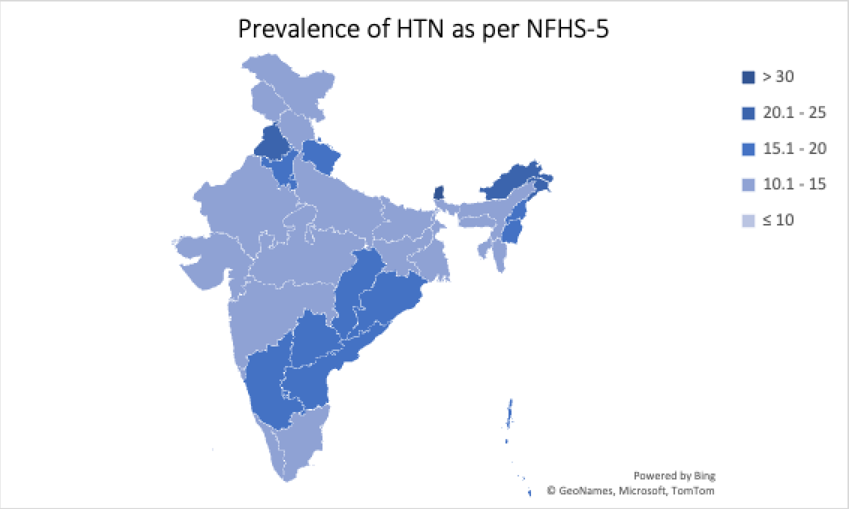

Figure 2 . Prevalence of HTN across states (% wise) across India.

Figure 3

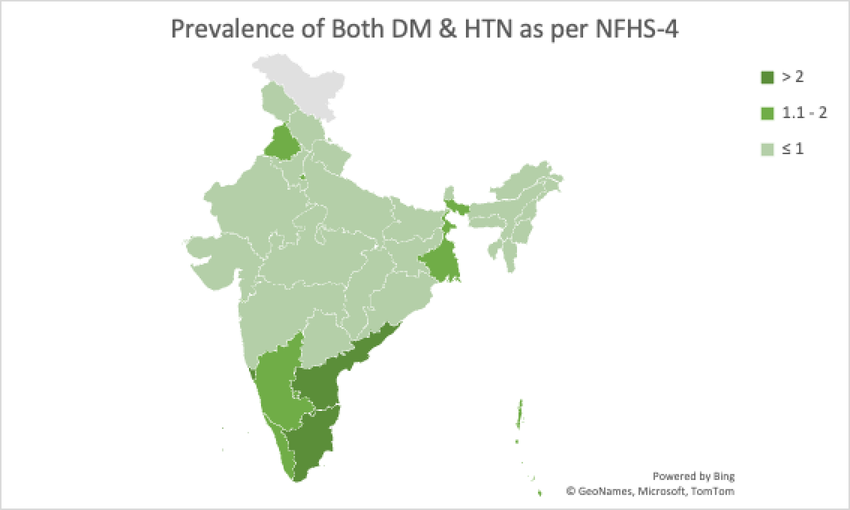

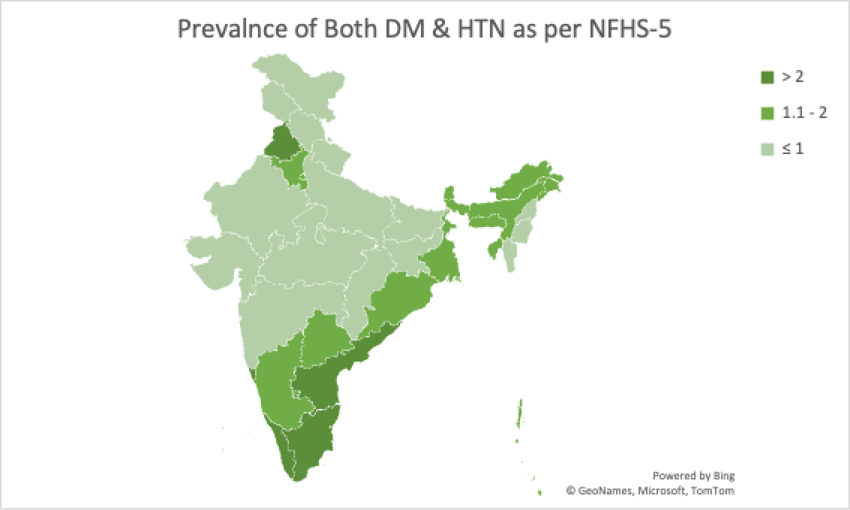

Figure 3. Prevalence of both DM and HTN in same individuals (% wise) across the states in India .

Click here to access/download
Supporting Information
Supplement.docx
