# Supplementary material for: Correlates of diabetes mellitus and hypertension in India: Change as evidenced from NFHS- 4 and 5 during 2015–2021
Source: PLoS One. 2024 Jul 18;19(7):e0305223. doi: 10.1371/journal.pone.0305223 (PMC11257250; doi:10.1371/journal.pone.0305223)
Supplement: Attachment — Submitted filename: Response to Reviewer.docx [file pone.0305223.s003.docx]

**Response to Reviewer -**

Thanks for reviewing our manuscript:

Comments from-

**Reviewer 1**. A line-by-line response has been detailed below.

| **Comments received** | **Change** | **Line no** | **Remarks/Response** |
| --- | --- | --- | --- |
| 1. Mention the brief methodology in the abstract as well. How was the data sourced can be mentioned. | Changed | 52 |  |
| 2. Start the sentences with words only and avoid numbers wherever possible. | Changed | 96-97 | Sentence has been reframed as per the suggestion |
| 3. The outline about the NFHS in the background section should be improved with respect to the writing style used. | Changed | 96-104 | Section has been rephrased |
| 4. The methodology should be restricted to the methods used in the current study only and not for NFHS or DLHS. The methodology used for the surveys can be given in the supplementary articles/appendix. | Changed | 132-136 | Methods section describing the details have been rewritten keeping it brief . |
| 5. Results and discussion are well written but would benefit for citation of some more recent references such as |  |  |  |
| a. Basu S, Maheshwari V, Gokalani R, Lahariya C. Prevalence and predictors of gestational diabetes mellitus and overt diabetes in pregnancy: A secondary analysis of nationwide data from India. Prev Med Res Rev 2024;1:52-8. DOI: 10.4103/PMRR. PMRR_11_23 b. Shivani, Sajjan1; Chawla, Parvinder S.1; Kalra, Khyati1. Is the Rule of Halves Still Relevant today? A Study from a Tertiary Care Hospital in India. Preventive Medicine Research & Reviews 1(3):p 134-136, May–Jun 2024. \| DOI: 10.4103/PMRR.PMRR_66_23 | changed but only one citation has been used of Gestational diabetes . | 352 | Other ref is only from a tertiary care hospital and it would not be justified to use that for a large data set . We have used a similar reference in national context |
| 6. The authors also need to contextualise the findings with Ayushman Bharat programs and packages under HWC on how these findings can be tackled through these programs and cite the relevant papers such as a. Lahariya C. Health & Wellness Centers to Strengthen Primary Health Care in India: Concept, Progress and Ways Forward. Indian J Pediatr. 2020; 87: 916-29. doi: 10.1007/s12098-020-03359-z. Epub 2020 Jul 8. b. Lahariya C. 'Ayushman Bharat' Program and Universal Health Coverage in India. Indian Pediatr. 2018; 55: 495-506. | Used and rewrote one section of the manuscript to utilise the references | 373-385 |  |

**Reviewer #2:** Dear Authors, the manuscript is meticulously written and has included all the required points. The paper has strong methodology and statistical approach. The data of NFHS 4 and NFHS 5 have been correlated in context to DM and HTN which seems to be very relevant.

**Response** – Thanks for the kind words and doing the review .
